# Improving Ambiguity Resolution for Medium Baselines Using Combined GPS and BDS Dual/Triple-Frequency Observations

**DOI:** 10.3390/s151127525

**Published:** 2015-10-30

**Authors:** Wang Gao, Chengfa Gao, Shuguo Pan, Denghui Wang, Jiadong Deng

**Affiliations:** 1 School of Transportation, Southeast University, Nanjing 210096, China; E-Mails: gaow@seu.edu.cn (W.G.); owenxun@gmail.com (D.W.); 220142542@seu.edu.cn (J.D.); 2 School of Instrument Science and Engineering, Southeast University, Nanjing 210096, China

**Keywords:** GPS/BDS, ambiguity resolution, partial ambiguity fixing, medium baselines, dual/triple-frequency observations

## Abstract

The regional constellation of the BeiDou navigation satellite system (BDS) has been providing continuous positioning, navigation and timing services since 27 December 2012, covering China and the surrounding area. Real-time kinematic (RTK) positioning with combined BDS and GPS observations is feasible. Besides, all satellites of BDS can transmit triple-frequency signals. Using the advantages of multi-pseudorange and carrier observations from multi-systems and multi-frequencies is expected to be of much benefit for ambiguity resolution (AR). We propose an integrated AR strategy for medium baselines by using the combined GPS and BDS dual/triple-frequency observations. In the method, firstly the extra-wide-lane (EWL) ambiguities of triple-frequency system, *i.e.*, BDS, are determined first. Then the dual-frequency WL ambiguities of BDS and GPS were resolved with the geometry-based model by using the BDS ambiguity-fixed EWL observations. After that, basic (*i.e.*, L1/L2 or B1/B2) ambiguities of BDS and GPS are estimated together with the so-called ionosphere-constrained model, where the ambiguity-fixed WL observations are added to enhance the model strength. During both of the WL and basic AR, a partial ambiguity fixing (PAF) strategy is adopted to weaken the negative influence of new-rising or low-elevation satellites. Experiments were conducted and presented, in which the GPS/BDS dual/triple-frequency data were collected in Nanjing and Zhengzhou of China, with the baseline distance varying from about 28.6 to 51.9 km. The results indicate that, compared to the single triple-frequency BDS system, the combined system can significantly enhance the AR model strength, and thus improve AR performance for medium baselines with a 75.7% reduction of initialization time on average. Besides, more accurate and stable positioning results can also be derived by using the combined GPS/BDS system.

## 1. Introduction

Besides the Global Navigation Satellite Systems (GNSS) GPS and GLONASS which have already been operational for a longer time, the Galileo and BeiDou navigation satellite system (BDS) have recently become operational. BDS officially announced its regional operation on 27 December 2012, and it is transmitting triple-frequency signals centred at B1 (1561.098 MHz), B2 (1207.140 MHz) and B3 (1268.520 MHz). Therefore, multi-system multi-frequency real-time kinematic (RTK) positioning is now feasible, which can enhance the geometric strength of the GNSS model [1,2,3,4]. The probability of correct ambiguity estimation [5,6,7] and the accuracy, availability and reliability of the precise positioning can be further improved by using the multi-frequency GNSS observations [8,9,10]. Fast and successful carrier-phase integer ambiguity resolution (IAR) is a prerequisite for RTK positioning, which is also factual in triple-frequency case. For the ambiguity resolution with triple-frequency observations, over the past two decades, many methods have been introduced. Forssell *et al.* [11] and Vollath *et al.* [12] proposed the three carrier ambiguity resolution (TCAR) approach. Hatch [13], Jung [14], Jung *et al.* [15] and Hatch *et al.* [16] proposed the cascading integer resolution (CIR) method, which was similar to TCAR. The early TCAR and CIR methods mainly estimate the integer ambiguities of the selected optimal combinations with a three-step rounding procedure. Teunissen *et al.* [17] compared TCAR, CIR and least-squares ambiguity decorrelation adjustment (LAMBDA) methods, pointing out that the early TCAR and CIR methods mainly use essentially the same geometry-free bootstrapping procedure, while LAMBDA is based on integer least squares with certain optimal properties. In the last ten years, Vollath [18], Feng and Rizos [19] and Hatch [20] extended the multiple-carrier ambiguity resolution to be suitable for the geometry-based model as well, which enhanced the model strength for AR, especially significant in the basic AR.

For medium or long baselines, the resolution of basic ambiguities is still a severe challenge, as atmospheric effects, especially the ionospheric effects, are non-ignorable for the basic (*i.e.*, L1/L2 or B1/B2) ambiguities, which just have wavelengths of usually less than 25 cm. Feng [5] selected three optimized virtual signals to reduce the ionospheric influence and resolved the ambiguity in a three-step procedure. Feng and Li [21] reported that ionospheric effects could be derived from the two ambiguity-fixed extra wide-lane (EWL) or wide-lane (WL) combinations, and then could be used to correct the ionospheric errors for basic AR. However because of the large amplified noises, some time is usually needed, *i.e.*, about 2 min to achieve the accurate ionospheric delay. Li *et al.* [6] used the ambiguity-fixed phase observations from two EWL or WL combinations instead of code observations to form geometry-free and ionospheric-free (GIF) linear combinations, allowing distance-independent ambiguity resolution. Wang and Rothacher [22] derived a combination that is similar to that in [6] for GPS, Galileo and COMPASS-III and analysed the characteristics of this combination. Zhang and He [23] tested with real BDS data and concluded that the GIF method was also influenced by the large noise amplification factor, and especially limited when multi-path errors exist in carrier phase observations.

Although triple-frequency or multi-frequency GNSS is the trend for almost all satellite systems, in the next many years, GNSS positioning users may have to deal with the mixture of dual-frequency and triple-frequency even quad-frequency observations, since it will take some years to finish the modernization of GPS or GLONASS. At present, some GPS or Galileo satellites can already transmit real triple-frequency observations, but only a small number of triple-frequency or multi-frequency satellites are available. In contrast, there are 14 BDS satellites in orbit, which all transmit triple-frequency signals, so a practical performance assessment of triple-frequency carrier AR algorithm is possible. Using real BDS observations, Tang *et al.* [24] compared the cascading rounding and integer least-square (ILS)-ased stepwise AR performance, and proposed a modified single-epoch stepwise AR which took the slant ionosphere effect of each satellite into consideration. Besides, the priori ionospheric information is used to enhance the AR model, which achieved a reliability of 96.56% for the 43 km baseline. However, the method is implemented for single-epoch mode, and the widely used ratio-test is not sufficiently reliable to check the AR performance in real-time practical applications. In this paper, we extend the single-epoch method in [24] to Kalman filtering mode. Moreover, the dual-frequency GPS and triple-frequency BDS observations are fused together to get a stronger AR model. Also, a partial ambiguity fixing (PAF) strategy is adopted to weaken the negative influence of new-rising or low-elevation satellites on AR. Real data with baseline distances varying from about 28.6 to 51.9 km, which all contain GPS/BDS dual/triple-frequency observations, are used to test the AR performance of the proposed method.

This paper is organized as follows: In Section 2 (Ambiguity resolution model), the EWL, WL and basic AR models are introduced, which includes the ionosphere-constrained model. Then in Section 3 (Partial ambiguity fixing strategy), a PAF method is introduced, which will be used in the fixing of WL and basic ambiguities. Real data with the baseline distance varying from about 28.6 to 51.9 km, which all contain GPS/BDS dual/triple-frequency observations, are processed in Section 4 (Experimental analysis) to test the performance of the proposed method. Finally in the last section the conclusions will be drawn.

## 2. Ambiguity Resolution Model

### 2.1. Basic Observation Equations and Definitions

Without loss of simplicity, the combined double-difference (DD) carrier phase and pseudorange observation equations in meters can be described by [5,6,24]:

(1) $$\Delta \phi_{\left(i,j,k\right)}=\Delta \rho +\Delta T-\eta_{\left(i,j,k\right)}\Delta I_{1}+\lambda_{\left(i,j,k\right)}\Delta N_{\left(i,j,k\right)}+\Delta \varepsilon_{\phi_{\left(i,j,k\right)}}$$

(2) $$\Delta P_{\left(i,j,k\right)}=\Delta \rho +\Delta T+\eta_{\left(i,j,k\right)}\Delta I_{1}\;\;+\Delta \varepsilon_{P_{\left(i,j,k\right)}}$$

where, the combined DD carrier phase $\Delta \phi_{\left(i,j,k\right)}$ and pseudorange $\Delta P_{\left(i,j,k\right)}$ are defined as:

(3) $$\;\;\Delta \phi_{\left(i,j,k\right)}=\frac{i\cdot f_{1}\cdot \Delta \phi_{1}+j\cdot f_{2}\cdot \Delta \phi_{2}+k\cdot f_{3}\cdot \Delta \phi_{3}}{i\cdot f_{1}+j\cdot f_{2}+k\cdot f_{3}}$$

(4) $$\Delta P_{\left(i,j,k\right)}=\frac{i\cdot f_{1}\cdot \Delta P_{1}+j\cdot f_{2}\cdot \Delta P_{2}+k\cdot f_{3}\cdot \Delta P_{3}}{i\cdot f_{1}+j\cdot f_{2}+k\cdot f_{3}}$$

In Equations (1)–(4), Δ is the double-difference (DD, station- and satellite-difference) operator; the symbol ρ represents geometric distance from satellite to receiver; T is the tropospheric delay; $I_{1}$ is the first-order ionospheric delay on BDS B1 or GPS L1 carrier (“first-order” will be omitted for brevity of notation below); i,j,k are integer coefficients; $\Delta \phi_{i}$ and $\Delta P_{i}$ represents the DD carrier and pseudorange measurement in distance for *i*th frequency $f_{i}$; ε represents the observation noise; the combined wavelength $\lambda_{\left(i,j,k\right)}$ and integer ambiguity $N_{\left(i,j,k\right)}$ are respectively defined as:

(5) $$\lambda_{\left(i,j,k\right)}=\frac{c}{i\cdot f_{1}+j\cdot f_{2}+k\cdot f_{3}}$$

(6) $$N_{\left(i,j,k\right)}=i\cdot N_{1}+j\cdot N_{2}+k\cdot N_{3}$$

The combined ionospheric scale factors, $\eta_{\left(i,j,k\right)}$ is defined as:

(7) $$\eta_{\left(i,j,k\right)}=\frac{f_{1}^{2}(i/f_{1}+j/f_{2}+k/f_{3})}{i\cdot f_{1}+j\cdot f_{2}+k\cdot f_{3}}$$

### 2.2. EWL and WL AR

The resolution of triple-frequency EWL ambiguities of BDS adopts the geometry-free and ionosphere-free model, given by Equation (8):

(8) $$\Delta N_{\left(0,-1,1\right)}=\left[\frac{\Delta \phi_{\left(0,-1,1\right)}-\Delta P_{\left(0,1,1\right)}}{\lambda_{\left(0,-1,1\right)}}\right]$$

where, [⋅] indicates the rounding operation, *i.e.*, the integer ambiguity $\Delta N_{\left(0,-1,1\right)}$ can be determined by rounding the float ambiguities to its nearest integer values. The model of Equation (8) has been verified to be simple and reliable by many researchers, and the performance using real BDS observations will be shown in Section 4.1 below.

After the EWL ambiguities are resolved successfully, the ambiguity-fixed EWL observations can be regarded as “pseudorange” observations to support the resolutions of WL ambiguities. Here the BDS/GPS combined geometry-based model is adopted to strengthen the AR model, where baseline components and the DD WL ambiguities of BDS and GPS are estimated, using Equation (9):

(9) $$\left[\begin{array}{c} v_{\mathrm{EWL}(0,-1,1),\;\;C}^{\prime }\\ v_{\mathrm{WL}(1,-1,0),\;\;C}\\ v_{\mathrm{WL}(1,-1),\;\;G} \end{array}\right]=\left[\begin{array}{ccc} B_{C} & 0 & 0\\ B_{C} & I\cdot \lambda_{(1,-1,0),C} & 0\\ B_{G} & 0 & I\cdot \lambda_{(1,-1),G} \end{array}\right]\left[\begin{array}{c} a\\ b_{C}\\ b_{G} \end{array}\right]-\left[\begin{array}{c} l_{\mathrm{EWL}(0,-1,1),C}^{\prime }\\ l_{\mathrm{WL}(1,-1,0),C}\\ l_{\mathrm{WL}(1,-1),\;\;G} \end{array}\right]$$

In Equation (9), the subscript ′C′ and ′G′ represent BDS and GPS respectively. $v_{\mathrm{EWL}(0,-1,1)}^{\prime }$ denotes the residual vector of ambiguity-fixed (0,−1,1) DD EWL observations of BDS. $v_{\mathrm{WL}(1,-1,0),\;\;C}$ and $v_{\mathrm{WL}(1,-1),\;\;G}$ denote the residual vectors of BDS (1,−1,0) DD and GPS (1,−1) DD WL observations. The matrix B is the design matrix of the baseline parameters and I is the identity matrix. The unknown parameters a and b are the vectors of baseline components and the DD carrier phase ambiguities, respectively. The last terms $l_{\mathrm{EWL}(0,-1,1),C}^{\prime }$, $l_{\mathrm{WL}(1,-1,0),C}$ and $l_{\mathrm{WL}(1,-1),\;\;G}$ are the observed minus computed (OMC) vectors of the corresponding observations, respectively.

The WL ambiguities can be searched and determined using the LAMBDA method thereby [25]. Here we fix the WL ambiguities of BDS firstly, as the EWL observations and WL observations of BDS have the same satellite geometry. Then the float WL ambiguities of GPS and corresponding variance-covariance matrix (vc-matrix) can be updated by using Equation (10) [26]:

(10) $$\begin{array}{l} {\tilde{b}}_{G}={\overset{˰}{b}}_{G}+Q_{{\overset{˰}{b}}_{G}{\overset{˰}{b}}_{C}}Q_{{\overset{˰}{b}}_{C}{\tilde{b}}_{C}}^{-1}({\overset{˰}{b}}_{C}-{\overset{˘}{b}}_{C})\\ Q_{{\tilde{b}}_{G}{\tilde{b}}_{G}}=Q_{{\overset{˰}{b}}_{G}{\overset{˰}{b}}_{G}}-Q_{{\overset{˰}{b}}_{G}{\overset{˰}{b}}_{C}}Q_{{\overset{˰}{b}}_{C}{\tilde{b}}_{C}}^{-1}Q_{{\overset{˰}{b}}_{C}{\overset{˰}{b}}_{G}} \end{array}$$

where $Q_{{\overset{˰}{b}}_{C}{\overset{˰}{b}}_{C}}$ and $Q_{{\overset{˰}{b}}_{G}{\overset{˰}{b}}_{G}}$ are the vc-matrix of ${\overset{˰}{b}}_{C}$ and ${\overset{˰}{b}}_{G}$ respectively, and $Q_{{\overset{˰}{b}}_{G}{\overset{˰}{b}}_{C}}$($Q_{{\overset{˰}{b}}_{C}{\overset{˰}{b}}_{G}}$) is the covariance matrix between ${\overset{˰}{b}}_{G}$(${\overset{˰}{b}}_{C}$) and ${\overset{˰}{b}}_{C}$(${\overset{˰}{b}}_{G}$); $Q_{{\tilde{b}}_{G}{\tilde{b}}_{G}}$ is the vc-matrix of updated GPS WL ambiguities, *i.e.*, ${\tilde{b}}_{G}$. One point to note here is that in the WL AR, the extreme low-elevation satellites may still suffer from considerable atmospheric effects, observation noises or other interferences. For this problem, we introduce a PAF strategy to adaptively choose the ambiguity subset, in which the ambiguities can be fixed reliably. The detailed expression of this strategy will be introduced in Section 3, below.

### 2.3. Basic AR with Ionosphere-Constrained Model

From the previous two steps, we get the EWL or WL ambiguities of BDS and GPS. These resolved WL ambiguities can be used to form the observation equations, together with the original carrier phase $\Delta \phi_{1}$. The observation equations, in which the baseline components, the ambiguities and the slant ionosphere delays in $\Delta \phi_{1}$ are unknown parameters, are:

(11) $$\left[\begin{array}{c} v_{\mathrm{WL}(1,-1,0),\;\;C}^{\prime }\\ v_{\mathrm{WL}(1,0,-1),\;\;C}^{\prime }\\ v_{B1,\;\;C}\\ v_{\mathrm{WL}(1,-1),\;\;\;G}^{\prime }\\ v_{L1,\;\;\;G} \end{array}\right]=\left[\begin{array}{ccccc} B_{C} & 0 & 0 & I\cdot f_{1,\;\;C}/f_{2,\;\;C} & 0\\ B_{C} & 0 & 0 & I\cdot f_{1,\;\;C}/f_{3,\;\;C} & 0\\ B_{C} & I\cdot \lambda_{1,\;\;C} & 0 & -I & 0\\ B_{G} & 0 & 0 & 0 & I\cdot f_{1,\;\;G}/f_{2,\;\;G}\\ B_{G} & 0 & I\cdot \lambda_{1,\;\;B} & 0 & -I \end{array}\right]\left[\begin{array}{c} a\\ b_{C}\\ b_{G}\\ \iota_{C}\\ \iota_{G} \end{array}\right]-\left[\begin{array}{c} l_{\mathrm{WL}(1,-1,0),\;\;C}^{\prime }\\ l_{\mathrm{WL}(1,0,-1),\;\;C}^{\prime }\\ l_{B1,\;\;\;C}\\ l_{\mathrm{WL}(1,-1),\;\;\;G}^{\prime }\\ l_{L1,\;\;G} \end{array}\right]$$

where $v_{B1,\;\;C}$ and $v_{L1,\;\;\;G}$ donate the residual vector of BDS B1 and GPS L1 carrier observations respectively. $l_{B1,\;\;C}$ and $l_{L1,\;\;\;G}$ are the corresponding OMC vectors. $\iota_{C}$ and $\iota_{G}$ are the vectors of ionosphere effects in L1 and B1 carrier observations of GPS and BDS. In Equation (11), the tropospheric delays are corrected by the high-precision GPT2 model [27]. Of course, one can easily extend Equation (11) to estimate the relative zenith tropospheric delay (RZTD) in the long baseline cases.

For Equation (11), we can work out some constraints to enhance the model strength. Here we treat the slant ionosphere effects to be a first-order Gauss-Markov process. The corresponding state translation equation between two consecutive epochs is:

(12) $$\iota_{k}=e^{-\frac{\Delta t}{\tau_{\iota }}}\;\;\iota_{k-1}+w_{\iota_{k}}\;,\;\;\;\;\;\;\;\sigma_{w_{\iota_{k}}}^{2}=\frac{\tau_{\iota }q_{\iota }}{2}(1-e^{-\frac{2\Delta t}{\tau_{\iota }}})$$

where Δt is time interval between two consecutive epochs; $\tau_{\iota }$ and $q_{\iota }$ are the correlation time and the spectrum density respectively. $w_{\iota_{k}}$ is the process white noise with variance $\sigma_{w_{\iota_{k}}}^{2}$. In the real-time RTK positioning, the sampling interval is very small relative to $\tau_{\iota }$ (in this study we take Δt=1 s), so the Equation (12) can be approximated to a random walk process:

(13) $$\iota_{k}=\iota_{k-1}+w_{\iota_{k}}\;,\;\;\;\;\;\;\;\sigma_{w_{\iota_{k}}}^{2}=q_{\iota }\Delta t$$

In the estimation of the DD slant ionosphere effects, we can also use some constraints, *i.e.*, the prior initial ionospheric bias $\iota_{k}^{0}$ with the corresponding covariance $\sigma_{\iota^{0}}^{2}$. According to research by Dach *et al.* [28], we can simply take $\iota_{k}^{0}=0$ with the baseline length up to 500 km. In this stage, it is rather crucial to specify the value of $\sigma_{\iota^{0}}$. As stated in [29,30], if $\sigma_{\iota^{0}}$ is too small, the model strength can, of course, be enhanced significantly, which however may result in a biased float solution; whereas if it is too large, the contribution of constraints to enhancing the model strength is downscaled, which could not be helpful to improving AR. Here we conservatively choose the large value of $\sigma_{\iota^{0}}$ to improve the float solution but without introducing bias.

For Equation (11), the standard Kalman filter based on the least squares criterion is adopted here to estimate the unknown parameters. In the filter, the baseline components and ambiguities are treated as constant-velocity process and time-invariant process respectively. For the slant ionospheric biases, referred to [30] and considering the practical results in Section 4 of this paper, we take $q_{\iota }=5{\text{ cm}}^{\text{2}}\text{/h}$, $\sigma_{\iota^{0}}=15\;\mathrm{cm}$ with $\iota_{k}^{0}=0$. Then in each epoch, the estimated unknowns ${\overset{˰}{x}}_{k}$ and the corresponding vc-matrix $Q_{{\overset{˰}{x}}_{k}}$ can be updated by the additional step as Equation (14):

(14) $$\begin{array}{l} {\tilde{x}}_{k}={\overset{˰}{x}}_{k}+Q_{{\overset{˰}{x}}_{k}{\overset{˰}{\iota }}_{k}}{\left(Q_{{\overset{˰}{\iota }}_{k}{\overset{˰}{\iota }}_{k}}+Q_{\iota_{k}^{0}\iota_{k}^{0}}\right)}^{-1}(\iota_{k}^{0}-{\overset{˰}{\iota }}_{k})\\ Q_{{\tilde{x}}_{k}{\tilde{x}}_{k}}=Q_{{\overset{˰}{x}}_{k}{\overset{˰}{x}}_{k}}-Q_{{\overset{˰}{x}}_{k}{\overset{˰}{\iota }}_{k}}{\left(Q_{{\overset{˰}{\iota }}_{k}{\overset{˰}{\iota }}_{k}}+Q_{\iota_{k}^{0}\iota_{k}^{0}}\right)}^{-1}Q_{{\overset{˰}{\iota }}_{k}{\overset{˰}{x}}_{k}} \end{array}$$

where, $Q_{{\overset{˰}{x}}_{k}{\overset{˰}{\iota }}_{k}}$ is the covariance matrix between all unknown parameters and the slant ionospheric parameters; $Q_{{\overset{˰}{\iota }}_{k}{\overset{˰}{\iota }}_{k}}$ is vc-matrix of slant ionospheric parameters; $Q_{\iota_{k}^{0}\iota_{k}^{0}}$ is the vc-matrix of the prior initial ionospheric bias $\iota_{k}^{0}$, which can be obtained by Equation (15):

(15) $$Q_{\iota_{k}^{0}\iota_{k}^{0}}=\sigma_{\iota^{0}}^{2}D_{m}^{T}D_{m}$$

where $D_{m}^{T}=[-e_{m}\;\;\;\;I_{m}]$ is the between-satellite single difference operator with the first satellite as reference. $e_{m}$ is m-column vector with all elements of 1. Similar to that in WL ambiguity resolution, there may be still some difficulties to reliably fix the ambiguities from low-elevation satellites. Also the PAF strategy which will be introduced in detail in the next section will be applied. Although the data is post-processed, the processing is completely analogous to the real-time processing, namely, the data loading and all computations are implemented epoch by epoch.

## 3. Partial Ambiguity Fixing Strategy

In the WL and basic ambiguity resolution described above, extreme low-elevation satellites may still suffer from considerable atmospheric effects, observation noises or other issues. It is therefore, for medium or long baselines, impossible to fix all ambiguities simultaneously sometimes. For this problem, we introduce a PAF strategy with ambiguity subset adaptively selected based on successively increased elevations. From the procedures described in Section 2.2 and Section 2.3, we can get the float ambiguities together with their corresponding vc-matrix. We can divide the ambiguities into two parts, of which the ones are assumed what can be reliably fixed, and the other ones are not or maybe not to be fixed reliably. As shown in Equation (16), we suppose that ${\overset{˰}{N}}_{a}$,$Q_{{\overset{˰}{N}}_{a}}$ and ${\overset{˰}{N}}_{b}$,$Q_{{\overset{˰}{N}}_{b}}$ are the ambiguities and the corresponding vc-matrix of the two parts respectively:

(16) $$\left[\begin{array}{c} {\overset{˰}{N}}_{a}\\ {\overset{˰}{N}}_{b} \end{array}\right]\cdot \left[\begin{array}{cc} Q_{{\overset{˰}{N}}_{a}} & Q_{{\overset{˰}{N}}_{a}{\overset{˰}{N}}_{b}}\\ Q_{{\overset{˰}{N}}_{b}{\overset{˰}{N}}_{a}} & Q_{{\overset{˰}{N}}_{b}} \end{array}\right]$$

If we can fix ${\overset{˰}{N}}_{a}$ reliably and the number of ambiguities in ${\overset{˰}{N}}_{a}$ is enough, we can directly use the fixed ambiguities in positioning calculations. Of course we can also use the fixed ambiguities to improve the accuracies of the remaining ambiguities and their vc-matrix [26]. In this paper we mainly consider the former case, that is to say we just fix the easy-fixed ambiguities as the available satellites are sufficient to the RTK positioning. However, the important thing is how to determine the subset. In this paper we get the subset by the following steps:

*Step (1)*: Sort the elevations of all satellites as an ascend order, and we can get the new elevation set like Equation (17):

(17) $$E=\left\{e_{1},e_{2},\cdots ,e_{n}\;\;|\;\;\;e_{1}<e_{2}<\cdots <e_{n}\;\right\}$$

where $e_{i}$ represents the elevation in the $i^{th}$ order.

*Step (2)*: Set the cut-off elevation $e_{c}$ at $e_{1}$, and we can get the ambiguity subset ${\overset{˰}{N}}_{a}(e_{1})$ (ambiguity subset with elevations no less than $e_{1}$) and its vc-matrix $Q_{{\overset{˰}{N}}_{a}}(e_{1})$, the corresponding elevations of which are larger than *e*_1_. Then the LAMBDA method is applied into the ambiguity search process. If the search results meet the following three conditions, the fixed ambiguities can be considered to pass the acceptance test and be used into the flowing positioning calculation.

(a) The bootstrapping AR success rate $P_{s}$, calculated according to Equation (18) from the decorrelated vc-matrix [31] is larger than the set threshold, $P_{0}$:

(18) $$P_{s}=\prod_{j=i}^{n}\left(2\Phi (\frac{1}{2\sigma_{{\overset{˰}{z}}_{j|J}}})-1\right)$$

where $\Phi (x)=\int_{-\infty }^{x}\frac{1}{\sqrt{2\pi }}\exp\{-\frac{1}{2}\nu^{2}\}d\nu$ and $\sigma_{{\overset{˰}{z}}_{j|J}}\;\;\;\;\;(\;\;j=i,\ldots ,n,\;\;\;J=\{j+1,\ldots ,n\}\;\;)$ denote the conditional standard deviations of the decorrelated ambiguities.

(b) The ratio of the second minimum quadratic form of integer ambiguities residuals and the minimum one [32,33], which is shown in Equation (19), is larger than the set threshold, $ratio_{0}$:

(19) $$ratio=\frac{||{\overset{˰}{N}}_{a}-{\overset{˘}{N}}_{a2}|{|}_{\;\;Q_{{\overset{˰}{N}}_{a}}}}{||{\overset{˰}{N}}_{a}-{\overset{˘}{N}}_{a1}|{|}_{\;\;Q_{{\overset{˰}{N}}_{a}}}}$$

where ${\overset{˘}{N}}_{a1}$ and ${\overset{˘}{N}}_{a2}$ are the ambiguity candidates with the minimum and second minimum quadratic form respectively; $\left||\cdot |{|}_{Q_{{\overset{˰}{N}}_{a}}}={\left(\cdot \right)}^{T}Q_{{\overset{˰}{N}}_{a}}^{-1}(\cdot \right)$.

(c) The number of ambiguities in the subset is larger than the set minimum threshold $n_{0}$ and the cut-off elevation is smaller than the set maximum threshold $e_{c0}$. These two conditions are set to ensure the selected satellites are still enough to get reliable positioning results, since too few or too high cut-off-elevation satellites are adverse to the positioning stability, especially for the vertical direction.

*Step (3)*: If the conditions in Step (2) cannot be met, the cut-off elevation will be set at $e_{2}$. Then Step (2) will be repeated. Of course if the cut-off elevation at $e_{2}$ is still unable to pass the acceptance test, the procedure will be continued with larger cut-off elevation. But if it cannot meet the condition (c) in Step (2), the circulation will be stopped and the current epoch keeps the ambiguities float.

In the experiments described later in Section 4, in WL AR $P_{0}$ is set at 0.90 considering the single-epoch mode; $ratio_{0}$, $n_{0}$ and $e_{c0}$ are set at 3.0, 5, and 35° respectively; in basic AR, $P_{0}$ is set at 0.99; $ratio_{0}$, $n_{0}$ and $e_{c0}$ are set at 2.0, 5, and 35°, respectively.

## 4. Experimental Analysis

In order to demonstrate the proposed method and evaluate its performance, a series of experiments were carried out using data collected in Nanjing (Jiangsu Province) and Zhengzhou (Henan Province), in China. These data all contain BDS triple-frequency and GPS dual-frequency pseudorange and carrier observations. Table 1 gives a summary of these data sets which include the baseline lengths, the observation dates, the observation duration, the sampling interval, and the corresponding observation location. During the data processing, the initial cut-off satellite elevation is set at 15°.

**Table 1:** Data Information.

No.	Length	Date	Duration	Interval	Location
A	28.6 km	6 May 2014	16 h	1 s	Nanjing
B	40.3 km	7 March 2014	11 h	1 s	Zhengzhou
C	51.9 km	7 March 2014	11 h	1 s	Zhengzhou

### 4.1. Results of EWL and WL AR

**Figure 1:**
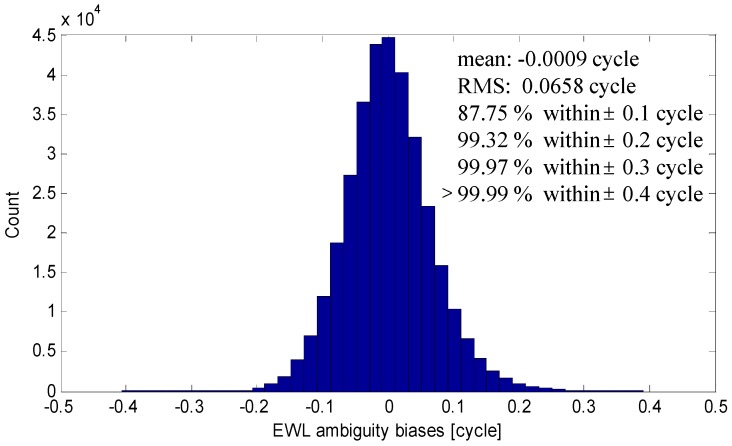
Results of (0, −1, 1) EWL AR.

The EWL and WL AR are implemented with single-epoch mode. We first investigate the single-epoch EWL AR performance. Figure 1 shows the biases of single-epoch (0, −1, 1) EWL AR of all BDS satellites in all epochs. The true ambiguities are verified by averaging the multi-epoch results. We can see almost all the biases are within ±0.4 cycle with the RMS of 0.0658 cycle. Over 99.97% of the biases are in the ±0.3 cycle, so we can reliably resolved the (0, −1, 1) ambiguities instantaneously. Besides, the (0, −1, 1) EWL AR is baseline-length independent, since it have eliminate the tropospheric error, ionospheric error and orbital errors, *etc*.

When using the LAMBDA method to search the WL integer ambiguities of BDS and GPS, we choose a stricter threshold of ratio, *i.e.*, 3.0, to ensure the enough reliability, since the WL ambiguities are solved with single-epoch model. Table 2 shows the statistical values, including the percentage of Ratio≥3.0 in FAF (full ambiguity fixing) and after PAF strategies, the percentage of PAF strategy (used) and the percentage of effective use of PAF. Here the effective use means the Ratio is larger than the threshold after the PAF strategy is used, while the FAF strategy cannot.

**Table 2:** Results of wide-lane ambiguity resolution with single epoch.

	WL AR of BDS ($\Delta N_{\left(1,-1,0\right)}$)			WL AR of GPS ($\Delta N_{\left(1,-1\right)}$)		
	A	B	C	A	B	C
Ratio≥3.0 of FAF (%)	100	100	98.6	100	>99.9	96.9
Ratio≥3.0 after PAF (%)	--	--	99.8	--	100	>99.9
PAF strategy used (%)	0	0	1.4	0	<0.1	3.1
Effective use of PAF	--	--	85.7	--	100	>96.8

From Table 2 we can see, for the two shorter baselines, *i.e.*, A and B, we almost get a 100% successful WL AR in single-epoch mode, and the PAF strategy is rarely used. However for the longest baseline, when using the FAF mode, we can just get 98.6% and 96.9% passed WL AR results for BDS and GPS, respectively. When the results cannot pass the set threshold, PAF mode will be implemented. We can see the percentages which pass the Ratio test will increase to 99.8% and over 99.9%. The percentages of effective use of PAF are 85.7% and over 96.8% respectively. In summary, from the above results we can see that when the PAF strategy is used, reliable WL ambiguities can be achieved almost instantaneously.

### 4.2. Results of Basic AR

**Figure 2:**
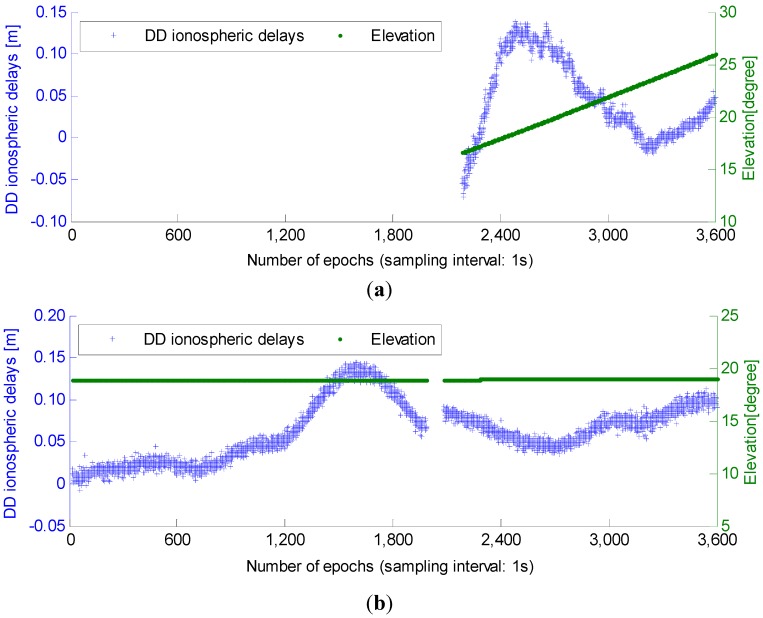
DD ionospheric delay and elevation variation of the two selected low-elevation satellites. (**a**) DD ionospheric delay and elevation variation of G25; (**b**) DD ionospheric delay and elevation variation of C05.

Before showing the results of basic AR, we first depict the DD ionospheric delays. Two low-elevation satellites (G25 and C05) in an hour from the 51.9 km baseline are selected to be analyzed. Figure 2 shows DD ionospheric delays, which are derived from the ambiguity-fixed geometry-free DD observations:

(20) $$\Delta I_{1}=\frac{f_{2}^{2}}{f_{1}^{2}-f_{2}^{2}}\cdot [\lambda_{1}\cdot (\Delta \varphi_{1}-\Delta N_{1})-\lambda_{2}\cdot (\Delta \varphi_{2}-\Delta N_{2})]$$

We can see from Figure 2 that the DD ionospheric delays reach a considerable magnitude, even larger than ten centimeters. This is the reason why ionospheric delays cannot be neglected in basic AR for medium baselines, and is also the reason that we choose $\sigma_{\iota }^{0}=15\;\mathrm{cm}$ in Section 2.3, avoiding biased float ambiguity solutions.

**Figure 3:**
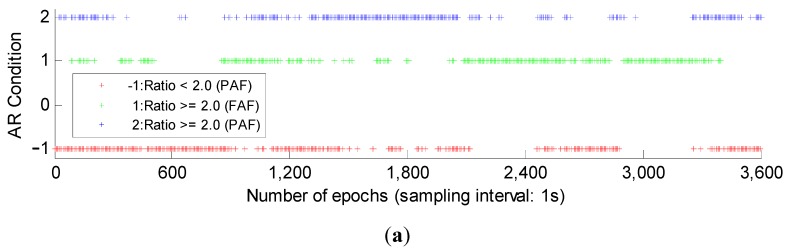

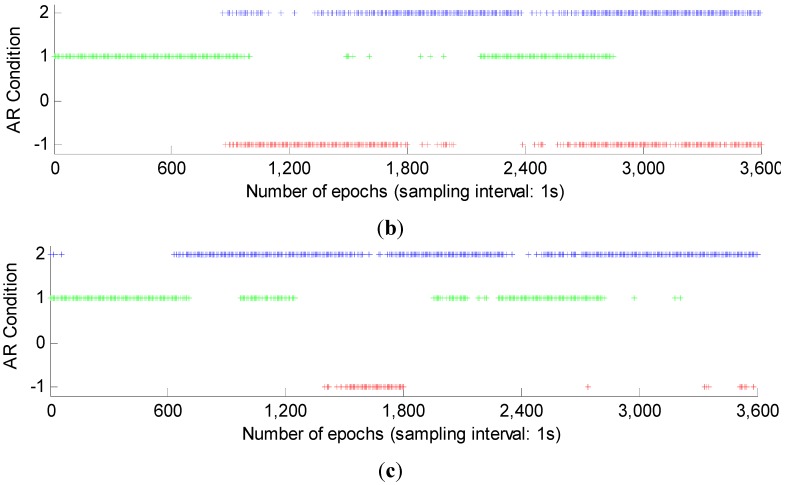
The single-epoch AR results of GPS, BDS and GPS/BDS combination. Three AR conditions, *i.e.*, “−1: Ratio < 2.0 (PAF)”, “1: Ratio ≥ 2.0 (FAF)” and “2: Ratio ≥ 2.0 (PAF)” are drawn in red, green and blue respectively. (**a**) single-epoch AR results of GPS; (**b**) single-epoch AR results of BDS; (**c**) single-epoch AR results of GPS/BDS combination.

Then we investigate the single-epoch basic AR performance. In the single-epoch AR, due to lack of geometry, the AR model has small success rate defined in Equation (17), *i.e.*, the weak model strength, so in the single-epoch case, the success rate will not be considered when using the PAF strategy. Figure 3 gives the single-epoch AR results, where three AR conditions, *i.e.*, “Ratio < 2.0 (PAF)”, “Ratio ≥ 2.0 (FAF)” and “Ratio ≥ 2.0 (PAF)” are displayed. The three conditions are drawn in red, green and blue respectively. The latter two conditions are seen as the index of successful AR when in the actual use. We can see although GPS/BDS combination improves the AR performance a lot compared with GPS or BDS, there are still some epochs failing to pass the acceptance test.

**Figure 4:**
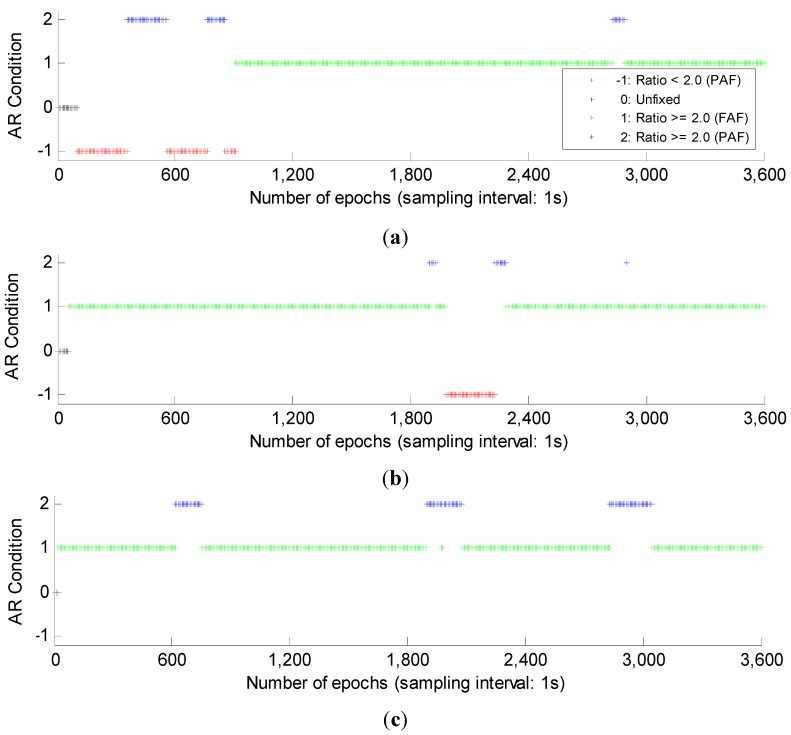
The filtering-based AR results of GPS, BDS and GPS/BDS combination. Four AR conditions, *i.e.*, “−1: Ratio <2.0 (PAF)”, “0: Unfixed”, “1: Ratio ≥ 2.0 (FAF)” and “2: Ratio ≥ 2.0 (PAF)” are drawn in red, black, green and blue respectively. (**a**) filtering-based AR results of GPS; (**b**) filtering-based AR results of BDS; (**c**) filtering-based AR results of GPS/BDS combination.

What’s more, strictly speaking, even if the AR in single-epoch mode get the same ratios with those in multi-epoch mode, the reliability is still much lower due to the much lower success rate [33]. The basic AR results using Kalman filtering are shown in Figure 4. Compared with the single-epoch mode, another AR condition “Unfixed” is introduced. This condition represents that in the initialization period in order to guarantee the AR reliability, the ambiguities will not be fixed until the success rate of FAF reaches 0.99. The four conditions are drawn in red, black, green and blue, respectively. We can see that using Kalman filtering, more successful AR can be obtained compared with the single-epoch mode. Especially with GPS/BDS combination, after the initialization, all epochs obtain the successful AR, while with single GPS or single BDS system, there are still some epochs cannot. This reflects the benefit of the GPS/BDS combination.

**Figure 5:**
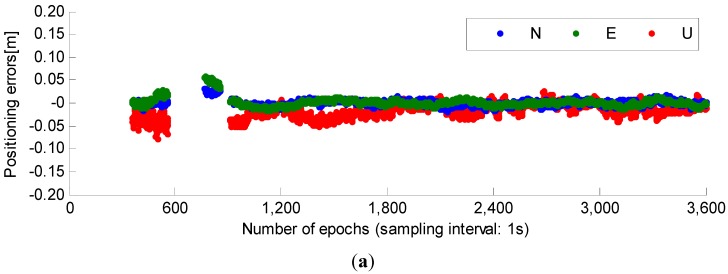

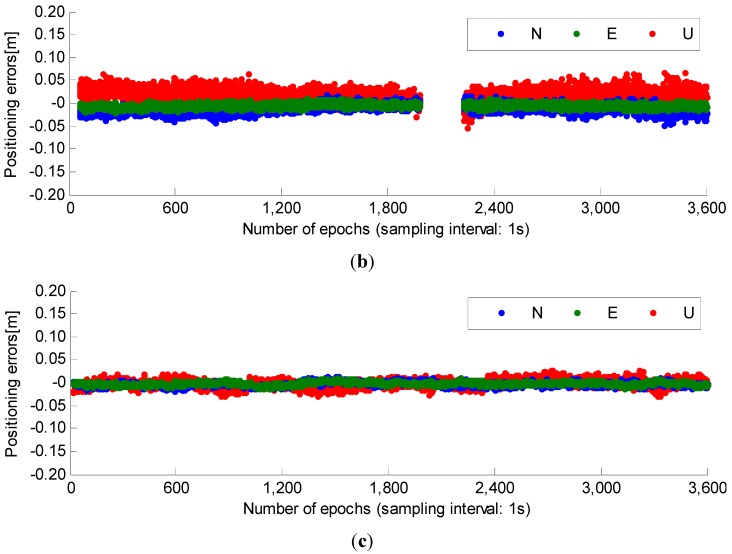
Positioning errors of GPS, BDS and GPS/BDS combination. (**a**) Positioning errors of GPS; (**b**) positioning errors of BDS; (**c**) positioning errors of GPS/BDS combination.

After fixing the ambiguities, the fixed basic and WL integer ambiguities can be back tracked into the ionosphere-free observations to calculate positioning results. The positioning biases of north, east and up (N/E/U for short) directions are shown in Figure 5. We can see with GPS/BDS combination, apart from more ambiguity-fixed epochs, more accurate and more stable positioning results can also be derived. This is another important benefit of the GPS/BDS combination.

From Figure 5, we also can see the positioning results of BDS have the largest instability. This is mainly caused by the satellite geometry, as the current BDS satellites mainly consist of GEO and IGSO satellites, so in China most visible satellites are in the south part of the skyplot. As more MEO satellites are launched step by step, the satellite geometry will no doubt become better.

**Figure 6:**
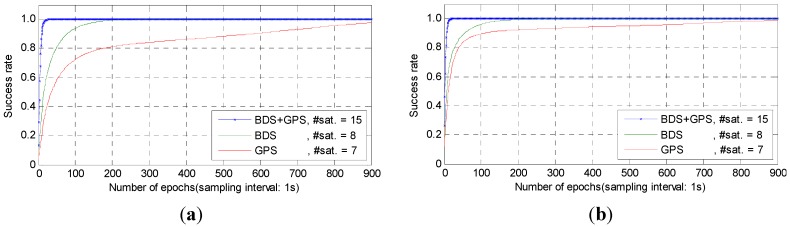
AR success rate of the selected initialized period. (**a**) AR success rate with non-ionosphere-constrained model; (**b**) AR success rate with ionosphere-constrained model.

As we all know, the initialization time is also very important in high-precision RTK positioning. In order to test the initialization time using the proposed method, each baseline in Table 1 is divided by half an hour in each period. The time-to-first-fix (TTFF), defined as the seconds needed for successful AR, is examined. Here, the successful AR is judged by both ratio test and success rate as criteria. The thresholds set in this paper are $P_{s}\geq 0.99$ and Ratio≥2.0. Figure 6 gives the AR success rate from a selected initialized period for example, where Figure 6a,b indicate the non-ionosphere-constrained and ionosphere-constrained results, respectively. From Figure 6a,b, we can see the success rate from the combined GPS/BDS systems is much higher than that from the single BDS or GPS. Besides, we can also see the ionosphere-constrained results are observably better than the non-ionosphere-constrained results. This also shows the significance of ionosphere-constrained model, which enhances the model strength especially in the initialized period.

We compute the average TTFF for both the ionosphere-constrained and non-ionosphere-constrained model. Because only dual-frequency observations are available for GPS in the experiments, for fairness, we just compare the TTFF from BDS and BDS/GPS combined systems. One point to note is that, before the first time that success rate of FAF reaches 0.99, the PAF strategy will not be applied. This is to avoid the useless computation in the initialization, in which serious ill-posedness generally exists in the normal equations [34,35]. Table 3 gives the average TTFF of each baseline. As we can see, for all the three baselines, the BDS/GPS combined system uses much less time than single BDS, about 75.7% reduction on average. This mainly benefits from the more redundancy provided by GPS, which evidently enhances the model strength. It needs to be noted here that, the TTFF in the table of baseline B baseline C and are almost the same. This is because the TTFF in this paper uses both ratio test and success rate as criteria, and in the experiments baseline B baseline C are collected in the same city and during the same periods, so the success rates show the same trend. In the definition of TTFF, actually, if we ignore the criteria of success rate and just consider the ratio test, the initialization time will drastically reduce. However it may be not reliable enough since the AR robustness has not been guaranteed.

**Table 3:** Results of narrow-lane ambiguity resolution.

	A		B		C	
	BDS	BDS + GPS	BDS	BDS + GPS	BDS	BDS + GPS
Average TTFF/s (non-ionosphere-constrained)	74.8	18.6	81.5	20.7	81.7	20.2
Average TTFF/s (ionosphere-constrained)	61.1	13.6	65.8	15.4	66.0	15.3

**Figure 7:**
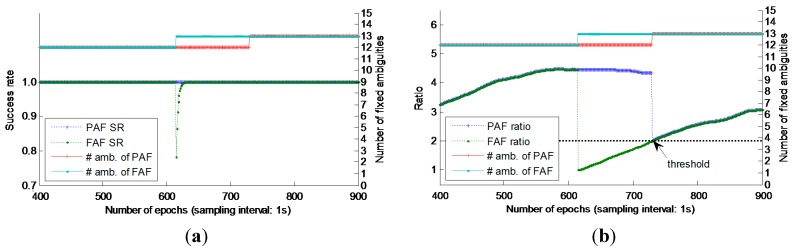
Basic AR performance using FAF and PAF strategy when a new-rising satellite appears. (**a**) AR success rate and the number of fixed ambiguities; (**b**) Ratio and the number of fixed ambiguities.

As mentioned above, after the first time that the success rate of FAF reaches 0.99, the PAF strategy will be applied to avoid the negative influence of low-elevation or new-rising satellite(s). In order to reveal how the PAF strategy works in detail, Figure 7 gives a typical case that a new-rising satellite appears, which includes the number of fixed ambiguities, success rate and ratio from both the FAF and PAF strategies. From Figure 7a,b, we can see when there has a new-rising satellite, both the success rate and ratio will drop dramatically under the thresholds, as the new-rising satellite suffers from the poor ambiguity precision and large ambiguity bias. When using PAF strategy, it can be solved well, since in AR search process, the new-rising satellite(s) will be abandoned adaptively by the set conditions or thresholds. In the later epochs, as the elevation increases and the ambiguity precision improves, the “new rising” ambiguity will be adopted in the fixed ambiguity subset. Although the ratio also drops a lot, it still meets the threshold, thus still ensure the AR reliability.

## 5. Conclusions

In this paper, we propose an integrated AR strategy by using the combined GPS and BDS dual/triple-frequency observations. Real GPS and BDS dual/triple-frequency observations are also processed to assess the improvement of the proposed method practically. The main conclusions are as follows:

(1) Using the combined GPS and BDS dual/triple-frequency observations can improve the AR performance significantly. The EWL ambiguities of BDS can be solved very well by using the triple-frequency observations instantaneously. Then the dual-frequency WL ambiguities of BDS and GPS can be also resolved reliably with the fused geometry-based model by using the BDS ambiguity-fixed EWL observations. Most importantly, the combined system observably enhances the model strength of basic AR compared with that in the single BDS system, of course the same for single GPS system. This improves the AR reliability and significantly shortens the initialization time positioning, about 75.7% reduction on average compared with a single third-frequency BDS system.
(2) The ionosphere-constrained model is proved useful to enhance the model strength, since the additional ionospheric constraints can be imposed. This especially contributes more in the beginning epochs, *i.e.*, the initialization period, in which serious ill-posedness exists in the normal equations.
(3) The PAF strategy was used both in WL and basic AR, which selects the ambiguity subset adaptively based on the successively increased elevations. It has been proved meaningful to weaken the negative influence of new-rising or low-elevation satellites.

## References

[1] Li X., Ge M., Dai X., Ren X., Fritsche M., Wickert J., Schuh H. (2015). Accuracy and reliability of multi-GNSS real-time precise positioning: GPS, GLONASS, BeiDou, and Galileo. J. Geod..

[2] Li X., Zhang X., Ren X., Fritsche M., Wickert J., Schuh H. (2015). Precise positioning with current multi-constellation Global Navigation Satellite Systems: GPS, GLONASS, Galileo and BeiDou. Sci. Rep..

[3] Odolinski R., Teunissen P.J.G., Odijk D. (2015). Combined BDS, Galileo, QZSS and GPS single-frequency RTK. GPS Solut..

[4] Han H., Wang J., Wang J., Tan X. (2015). Performance Analysis on Carrier Phase-Based Tightly-Coupled GPS/BDS/INS Integration in GNSS Degraded and Denied Environments. Sensors.

[5] Feng Y. (2008). GNSS three carrier ambiguity resolution using ionosphere-reduced virtual signals. J. Geod..

[6] Li B., Feng Y., Shen Y. (2010). Three carrier ambiguity resolution: Distance-independent performance demonstrated using semi-generated triple frequency GPS signals. GPS Solut..

[7] Li B., Shen Y., Zhang X. (2013). Three frequency GNSS navigation prospect demonstrated with semi-simulated data. Adv. Space Res..

[8] Yang Y., Li J., Xu J., Tang J., Guo H., He H. (2011). Contribution of the compass satellite navigation system to global PNT users. Chin. Sci. Bull..

[9] He H., Li J., Yang Y., Xu J., Guo H., Wang A. (2014). Performance assessment of single-and dual-frequency BeiDou/GPS single-epoch kinematic positioning. GPS Solut..

[10] Li J., Yang Y., Xu J., He H., Guo H. (2015). GNSS multi-carrier fast partial ambiguity resolution strategy tested with real BDS/GPS dual-and triple-frequency observations. GPS Solut..

[11] Forssell B., Martin-Neira M., Harrisz R.A. Carrier phase ambiguity resolution in GNSS-2. Proceedings of the 10th International Technical Meeting of the Satellite Division of the Institute of Navigation (ION GPS 1997).

[12] Vollath U., Birnbach S., Landau L., Fraile-Ordoñez J.M., Martin-Neira M. (1999). Analysis of Three-Carrier Ambiguity Resolution Technique for Precise Relative Positioning in GNSS-2. Navigation.

[13] Hatch R. (1996). The promise of a third frequency. GPS World.

[14] Jung J. High integrity carrier phase navigation for future LAAS using multiple civilian GPS signals. Proceedings of the 12th International Technical Meeting of the Satellite Division of the Institute of Navigation (ION GPS 1999).

[15] Jung J., Enge P., Pervan B. Optimization of cascade integer resolution with three civil GPS frequencies. Proceedings of the 13th International Technical Meeting of the Satellite Division of the Institute of Navigation (ION GPS 2000).

[16] Hatch R., Jung J., Enge P., Pervan B. (2000). Civilian GPS: The benefits of three frequencies. GPS Solut..

[17] Teunissen P.J.G., Joosten P., Tiberius C. A comparison of TCAR, CIR and LAMBDA GNSS ambiguity resolution. Proceedings of the 15th International Technical Meeting of the Satellite Division of the Institute of Navigation (ION GPS 2002).

[18] Vollath U. The factorized multi-carrier ambiguity resolution (FAMCAR) approach for efficient carrier-phase ambiguity estimation. Proceedings of the 17th International Technical Meeting of the Satellite Division of the Institute of Navigation (ION GNSS 2004).

[19] Feng Y., Rizos C. Three carrier approaches for future global, regional and local GNSS positioning services: Concepts and performance perspectives. Proceedings of the 18th International Technical Meeting of the Satellite Division of the Institute of Navigation (ION GNSS 2005).

[20] Hatch R. A new three-frequency, geometry-free technique for ambiguity resolution. Proceedings of the 19th International Technical Meeting of the Satellite Division of the Institute of Navigation (ION GNSS 2006).

[21] Feng Y., Li B. (2008). A benefit of multiple carrier GNSS signals: Regional scale network-based RTK with doubled inter-station distances. J. Spat. Sci..

[22] Wang K., Rothacher M. (2013). Ambiguity resolution for triple-frequency geometry-free and ionosphere-free combination tested with real data. J. Geod..

[23] Zhang X., He X. (2015). Performance analysis of triple-frequency ambiguity resolution with BeiDou observations. GPS Solut..

[24] Tang W., Deng C., Shi C., Liu J. (2014). Triple-frequency carrier ambiguity resolution for Beidou navigation satellite system. GPS Solut..

[25] Teunissen P.J.G. (1995). The least-squares ambiguity decorrelation adjustment: A method for fast GPS integer ambiguity estimation. J. Geod..

[26] Teunissen P.J.G., Joosten P., Tiberius C. Geometry-free ambiguity success rates in case of partial fixing. Proceedings of the 1999 National Technical Meeting of The Institute of Navigation (ION-NTM 1999).

[27] Böhm J., Möller G., Schindelegger M., Pain G., Weber R. (2015). Development of an improved empirical model for slant delays in the troposphere (GPT2w). GPS Solut..

[28] Dach R., Hugentobler U., Fridez P., Meindl M. (2007). Bernese GPS Software.

[29] Odijk D. Weighting ionospheric corrections to improve fast GPS positioning over medium distances. Proceedings of the 13th International Technical Meeting of the Satellite Division of the Institute of Navigation (ION GPS 2000).

[30] Li B., Shen Y., Feng Y., Gao W., Yang L. (2014). GNSS ambiguity resolution with controllable failure rate for long baseline network RTK. J. Geod..

[31] Teunissen P.J.G. (1998). Success probability of integer GPS ambiguity rounding and bootstrapping. J. Geod..

[32] Teunissen P.J.G., Verhagen S. (2009). The GNSS ambiguity ratio-test revisited: A better way of using it. Surv. Rev..

[33] Verhagen S., Teunissen P.J.G. (2013). The ratio test for future GNSS ambiguity resolution. GPS Solut..

[34] Li B., Shen Y., Feng Y. (2010). Fast GNSS ambiguity resolution as an ill-posed problem. J. Geod..

[35] Pan S., Gao W., Wang S., Meng X., Wang Q. (2014). Analysis of ill posedness in double differential ambiguity resolution of BDS. Surv. Rev..

